# *Viscum coloratum* (Komar.) Nakai: A Review of Botany, Phytochemistry, Pharmacology, Pharmacokinetics and Toxicology

**DOI:** 10.3390/biom15070974

**Published:** 2025-07-07

**Authors:** Han Di, Congcong Shen, Shengyu Zhang, Yanhong Wang, Feng Guan

**Affiliations:** 1 School of Pharmacy, Heilongjiang University of Chinese Medicine, Harbin 150040, China; h0519333@gmail.com (H.D.); shencongcong66@gmail.com (C.S.); 376713942tt@gmail.com (S.Z.); 2 Key Laboratory of Basic and Application Research of Beiyao, Ministry of Education, Heilongjiang University of Chinese Medicine, Harbin 150040, China

**Keywords:** *Viscum coloratum* (Komar.) Nakai, phytochemistry, anti-inflammatory effect, anticancer effect, pharmacokinetics, toxicology

## Abstract

*Viscum coloratum* (Komar.) Nakai (*V. coloratum*) is a traditional Chinese herbal medicine. It is used in treating rheumatism and paralysis, lumbar and knee soreness, weakness of the muscles and bones, excessive leakage of menstruation, leakage of blood in pregnancy, restlessness of the fetus, dizziness and vertigo. All information about *V. coloratum* was collected through databases such as PubMed, Google Scholar, Web of Science, and the China National Knowledge Infrastructure and supplemented by consulting classical Chinese medical books. To date, 111 compounds have been isolated and identified from *V. coloratum*, including flavonoids, phenylpropanoids, terpenoids, diarylheptanoids, alkaloids, other components, and macromolecular compounds, such as polysaccharides and lectins. These chemical components exhibit anti-inflammatory, anticancer, antioxidant, and anti-cardiovascular disease effects, among other beneficial effects. According to the reports, alkaloids, lectins, and other chemical components present in *V. coloratum* may induce toxicity due to excessive intake or accidental ingestion. However, there are few reports on the toxicology of *V. coloratum*, and there is a lack of studies on the toxicity of *V. coloratum* with known in vitro or preclinical activity. It is suggested that further studies on the toxicology of *V. coloratum* should be conducted in the future. In this paper, the botany, traditional uses, phytochemistry, pharmacology, and pharmacokinetics of *V. coloratum* are summarized, and the progress and shortcomings in toxicology are discussed, so as to provide a possible direction for future research on *V. coloratum*.

## 1. Introduction

*V. coloratum* is a hemiparasitic plant in the Loranthaceae family [1]. It is usually called Hujisheng in China. Its medicinal parts are dried stems and branches with leaves. It is bitter in flavor and neutral in nature, pertains to the Liver Meridian and the Kidney Meridian in traditional Chinese medicine theory, and is mainly used for lumbar and knee pain, rheumatism and paralysis, fetal restlessness, and blood leakage from the fetus [2]. *V. coloratum* has a long history of medicinal use since ancient times [3]. *V. coloratum* was first recorded as Sangshangjisheng in the ancient Chinese medical book *Shennong’s Classic of Materia Medica*, which means that it was originally found parasitic on mulberry trees [4]. In the records of *MingYi BieLu* of the Northern and Southern dynasties, the medicinal value of *V. coloratum* was gradually revealed, such as treating wounds and relieving pain [5]. It was pointed out that *V. coloratum* mainly grew on mulberry trees. *V. coloratum* was included in the 2020 edition of the *Pharmacopoeia of the People’s Republic of China* (ChP, 2020) [2]. It is of great medicinal importance in many countries around the world [6]. Particularly in Europe, it has gained significant attention in both traditional medicine and modern alternative therapies. For example, Germany and Switzerland have approved its use in adjunctive antitumor therapy [7]. Additionally, it plays an important role in immune regulation in countries such as Japan and South Korea [8].

There are approximately 70 species of *Viscum*, of which 11 species and 1 variety are found in China and distributed in most provinces. The *Pharmacopoeia of the People’s Republic of* China contains only one species, *V*. *coloratum*, which currently serves as the predominant medicinal species in the marketplace [5]. There are two varieties of plants depending on the color of the fruit, *Viscum coloratum* f. *lutescens* and *Viscum coloratum* f. *rubroaurantiacum*, which are equally medicinal [9]. *Viscum album* L (*V. album* L.) is mostly produced in Europe and has been included in the pharmacopoeias of many European countries [10]; it is also known as Oujisheng in China.

Medicinal plants are an important therapeutic resource and hold significant value in medicine. Valued for their mild yet effective therapeutic actions and low incidence of adverse reactions, they are widely used clinically, playing important roles in areas such as cancer prevention [11] and inflammation inhibition [12]. *V*. *coloratum*, as a notable medicinal plant, has gained significant attention due to its rich chemical constituents and unique pharmacological effects. For decades, *V. coloratum* has been extensively studied. Phytochemical studies show that there are many active components in *V. coloratum* [13]. At present, 111 compounds have been isolated, identified and characterized, which are mainly divided into flavonoids [14], phenylpropanoids [15], terpenoids [16], diphenylheptanes [17], alkaloids [18], polysaccharides [19], lectins, etc. [20]. Among them, flavonoids and phenylpropanoids account for the largest proportion, representing ca. 54.4% of all compounds. A wide range of biological activities has been demonstrated in these compounds [21], such as anticancer effect, anti-inflammatory effect, antiviral effect, and antioxidant effect, and they are clinically used in rheumatoid arthritis, inflammatory bowel disease, lung cancer, arrhythmia, etc. [22].

Researchers have identified the complete chloroplast (CP) genome of *V. coloratum.* Moreover, 30 shared CP genomes of 12 species, including *V. album* L., are analyzed through phylogenetic analysis, and evolutionary trees are constructed using maximum likelihood (ML) methods. The results show that *V. coloratum* is most closely related to the European species *V. album* L. [23]. The existing studies show that lectins in *V. album* L. can exhibit significant toxicity by inhibiting protein aggregation through a mechanism of action highly similar to that of ricin [24]. However, the toxicity characteristics and molecular mechanism of *V. coloratum*, a sister species of *V. album* L., have not been systematically studied. In the current literature, toxicology studies of *V. coloratum* are mostly limited to the preliminary characterization of the crude extract, and the activities, targets, and dosing relationships of its specific toxic components are not clear. In view of the potential application of *V. coloratum* in traditional medicine and modern tumor therapy, it is of great clinical significance to analyze the mechanism of toxicity of *V. coloratum*.

## 2. Botany

**Figure 1:**
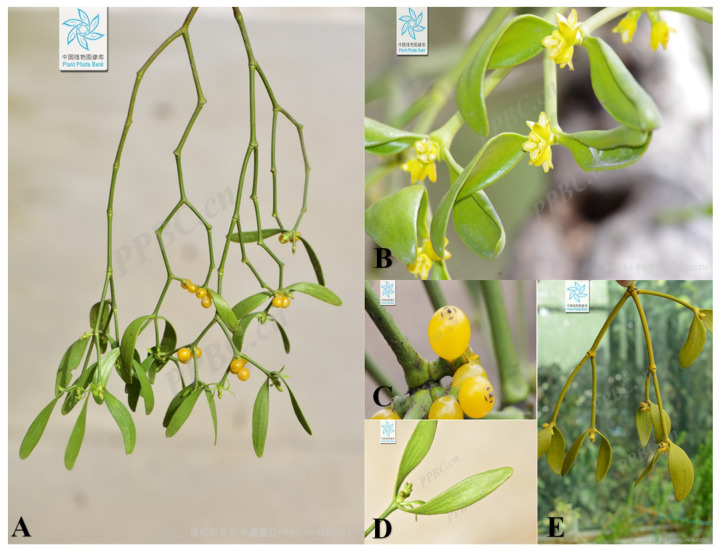
Plant morphology of *V. coloratum*. (**A**) The whole plant of *V. coloratum*, (**B**) flowers, (**C**) fruits, (**D**) leaves, (**E**) branches and stems. The image of *V. coloratum* is from the Plant Photo Bank of China.

*V. coloratum* is a hemiparasitic plant [25]. It can carry out photosynthesis by itself, and its roots can be specialized into parasitic roots [26], so that it can directly connect with the xylem ducts of the host and obtain water and inorganic salts from it [27]. The main way of living for *V. coloratum* is as a single cluster, and it is abundant on sunny slopes and flat mountain slopes and in secondary forests around the farmland. According to the World Flora Online Records (www.worldfloraonline.org, accessed on 15 April 2025), *V. coloratum* is a shrub ranging from 0.3 to 0.8 m in height. Its cylindrical stems and branches exhibit sparse branching patterns, typically dividing dichotomously or trichotomously. The nodes are slightly swollen. The internodes of the small branches measure 5–10 cm in length and 3–5 mm in thickness, while the branches exhibit irregular wrinkles on their undersides. The leaves are opposite, occasionally whorled in groups of three, thickly coriaceous or coriaceous, oblong to elliptic-lanceolate, 3–7 cm long and 0.7–1.5 (–2) cm wide, with a rounded to obtuse apex and attenuate base. The leaves possess 3–5 basal veins, and the petioles are short. The plant is dioecious. Inflorescences are terminal or axillary at dichotomous branch nodes. Male inflorescences are cymose, the peduncle is nearly absent or up to 5 mm long, the involucre is navicular, 5–7 mm long, typically bearing 3 flowers, and the central flower has 2 bracts or is ebracteate. Male flowers are ovoid in the bud, 3–4 mm long, with 4 sepals, ovate. The anthers are elliptic, 2.5–3 mm long. The female inflorescences are in the form of clustered spikes, with total pedicels 2–3 mm long or rarely absent, with 3–5 flowers, terminal flowers with 2 bracts or absent, interspersed flowers with 1 bract each. Bracts are broadly triangular, ca. 1.5 mm long, initially with fine marginal hairs, later becoming entirely hairy. Female flowers are ovoid at the bud stage and measure ca. 2 mm long. The receptacle is long oval-shaped. There are 4 sepals, triangular, ca. 1 mm long. The stigma is papillate. The fruits are globose, 6–8 mm in diameter, with persistent styles; mature fruits are pale yellow to orange-red, bearing a smooth rind. The flowering period is from April to May, and the fruiting period is from September to November. The characteristics of the plant are shown in Figure 1 (https://ppbc.iplant.cn, accessed on 17 April 2025). In China, *V. coloratum* is mainly distributed in Guangxi Zhuang Autonomous Region, Gansu Province, Fujian Province, Zhejiang Province, Guizhou Province, and other regions, except Xinjiang, Tibet, Yunnan, and Guangdong. Outside China, it is found in the Russian Far East, North Korea, Japan, etc. [28]. Additionally, *V. coloratum* has two synonyms: *Viscum album* var. *coloratum* (Kom.) Ohwi (primarily distributed in South Korea, Japan, and North Korea) and *Viscum album* subsp. *coloratum* Kom. (mainly found in Japan).

## 3. Traditional Uses

*V. coloratum* has a long history of medicinal use in China [29]. Its medicinal value for treating wounds and relieving arthralgia was first described in the *MingYi BieLu* of the Northern and Southern dynasties. In the Northern and Southern dynasties, the *Notes on the Book of Bencao Jing Jizhu* described in detail the morphology of parasites on mulberry for the first time, and its characteristics were consistent with the morphology of *V. coloratum* in modern botany, providing key evidence for confirming its identity. *Xinxiu Bencao* of the Tang Dynasty not only documented the morphology and ecological habits of *V. coloratum*, but also described its symbiotic relationship with birds that facilitate seed dispersal, with the name Hujisheng appearing for the first time. The *Compendium of Materia Medica* and *Bencao Yuanshi* of the Ming Dynasty described it as a remedy for lumbar pain, capable of benefiting the kidneys, strengthening the backs of children, enriching the skin, firming the hair and teeth, growing the beard and eyebrows, and stabilizing the fetus [30]. *Bencao Beiyao* and *Bencao Congxin* of the Qing Dynasty recorded that it helps the muscles and bones and fixes the teeth and hair, disperses wind-dampness, sweetens and benefits the blood, stops the leakage of blood, stops lactation, settles the fetus, disperses sores in the surgical field, and chases the wind-dampness. In modern clinical practice, it mainly follows the therapeutic principle of compound coordination of the traditional Chinese medicine and forms a multi-target regulatory system through compatibility with other medicinal materials, rather than application of a single medicine [31]. Its compatibility strategy strictly follows the theory of monarch, minister, assistant, and envoy, for example, Qufeng zhitong tablets, Jiangyaping tablets, Shujin huoxue tablets, *Angelica* parasitic fluid, etc. In addition, in some northeastern parts of China, *V. coloratum* is one of the rare evergreen plants [32]. Especially in the early spring season in northeastern China, this evergreen shrub is an ornamental plant because it retains its leaves fat and green in the extremely cold environment of −30 °C.

## 4. Phytochemistry

More than 110 compounds have been isolated and identified from *V. coloratum*, including flavonoids, phenylpropanoids, terpenoids, alkaloids, polysaccharides, and lectins. Among them, flavonoids account for ca. 35% of the total compounds, phenylpropanoids account for ca. 19% of the total compounds, and terpenoids account for ca. 18%, indicating that these three types of compounds are the main active components of *V. coloratum*. In addition, we can find that flavonoids, phenylpropanoids, and terpenoids play an important role in the treatment of *V. coloratum*. The identified chemical components are summarized in Table 1.

### 4.1. Flavonoids

**Figure 2:**
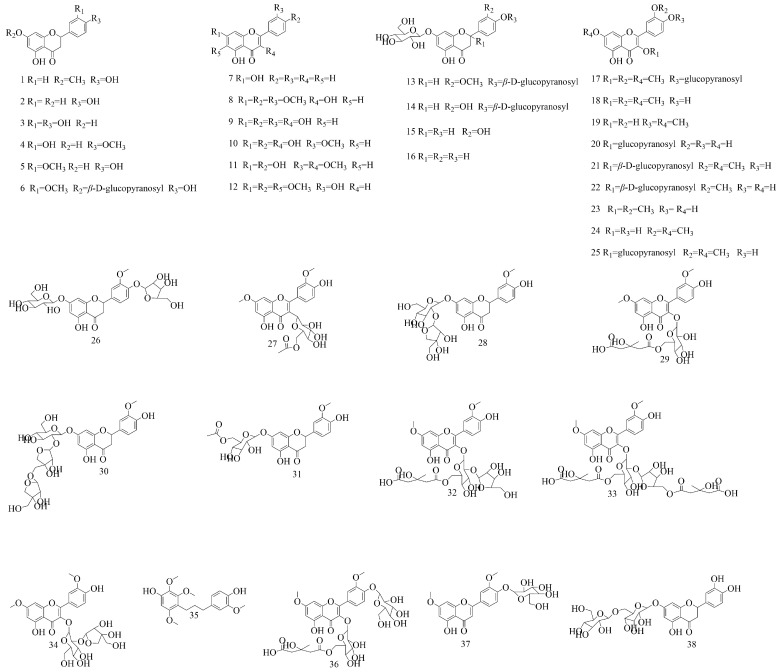
The structures of flavonoids in *V. coloratum*.

Flavonoids are a kind of compound widely existing in nature, with a wide variety of biological activities. Their main parent nuclei are 2-phenylchromogens, most of which have a C6–C3–C6 structure [49]. Currently, 38 kinds of flavonoids, including flavones, flavonols, and dihydroflavonoids, have been isolated and identified in *V. coloratum.* The content of flavonoids in *V. coloratum* is relatively abundant, such as homoeriodictyol, viscumneoside (**26~34**), etc. These flavonoids have multiple pharmacological effects [50], such as antioxidant, anti-inflammatory, and antitumor, and are recognized as pivotal mediators of the pharmacological effects of *V. coloratum*. The structures of these flavonoids from **1** to **38** are shown in Figure 2.

### 4.2. Phenylpropanoids

**Figure 3:**
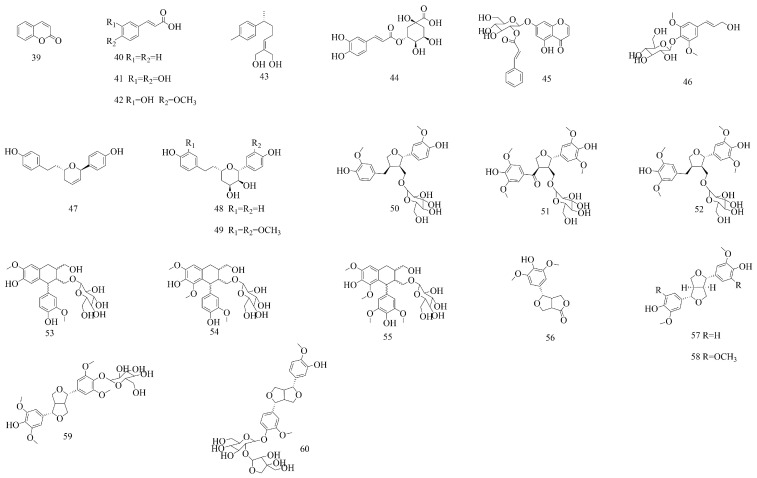
The structures of phenylpropanoids in *V. coloratum*.

Phenylpropanoids are a class of natural components with one or more C6–C3 units [51]. More than 20 kinds of phenylpropanoids have been isolated and identified, including coumarins, simple phenylpropanoids, and lignans. Lignans are the main subclass of phenylpropanoids. In addition, the content of syringin (**46**) in *V. coloratum*, as stipulated in the ChP, 2020, shall not be less than 0.040%. It has been found that compounds such as syringaresinol-O-*β*-D-glucopyranoside (**59**) in *V. coloratum* can be converted into hormone-like mammalian derivatives through hormone conversion and play their estrogenic effects through binding with estrogen receptors (ERs) or by competing with internal estrogen for ERs, thereby inhibiting the effect on bone resorption [15]. This can provide a strong basis for future application in the development of *V. coloratum* as an osteoporosis drug. Their structures are shown in Figure 3.

### 4.3. Diphenylheptanes

**Figure 4:**
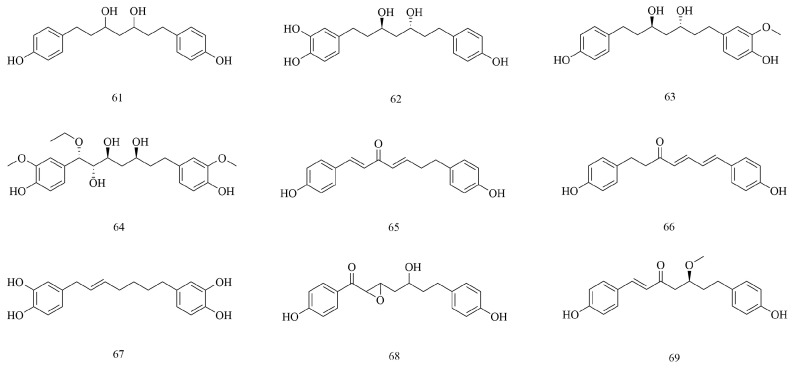
The structures of diphenylheptanes in *V. coloratum*.

Diphenylheptanes are a class of compounds with 1,7-disubstituted phenyl groups and heptane as the skeleton structure [52]. According to the straight-chain or the ring structure of the diphenyl group, they can be classified into two categories: chain diphenylheptanes and macrocyclic diphenylheptanes. Chain diphenylheptanes can be further classified into acyclic straight-chain diphenylheptanes and epoxy diphenylheptanes according to whether the seven carbons on the alkane structure are in the oxygen ring. At present, nine kinds of diphenylheptanes (**61–69**) have been isolated and identified in *V. coloratum*; for example, diphenylheptane C (**65**) and diphenylheptane B (**67**) are chain diphenylheptanes. It was found that mistletonone (**68**) showed a significant scavenging effect on hydroxyl radicals and superoxide anion radicals in direct determination [17]. *V. coloratum* exhibits potent antioxidant activity, and diphenylheptane structures are shown in Figure 4.

### 4.4. Terpenoids

**Figure 5:**
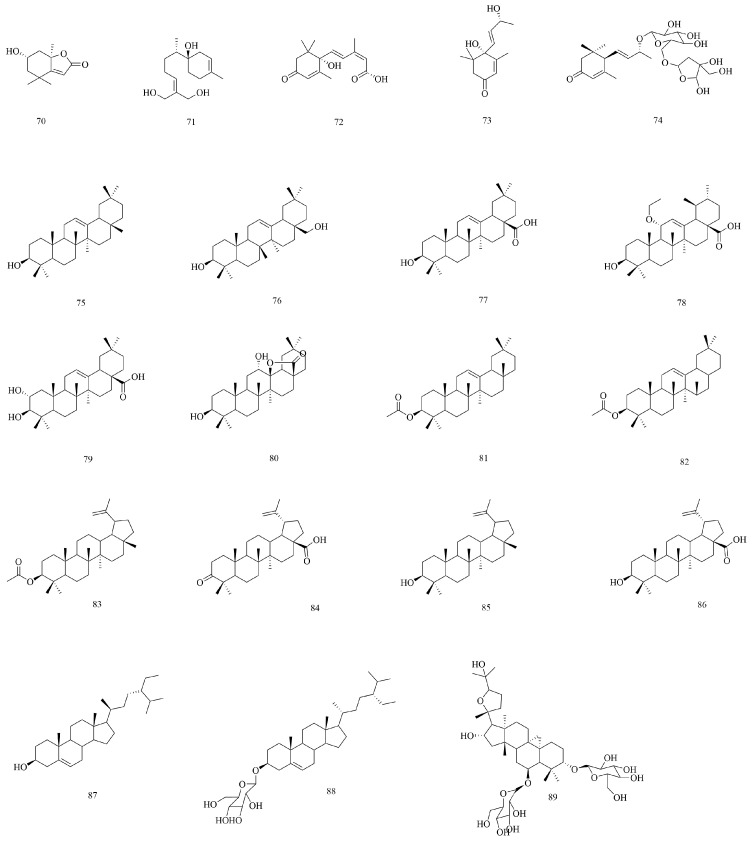
The structures of terpenoids in *V. coloratum*.

Terpenoids are derived from methylpentanedihydroxy acid and have two or more isoprene structures in their basic skeleton [53]. At present, 20 kinds of terpenoids have been isolated and identified, such as sesquiterpenes, triterpenes, etc. Among the known terpenoids, triterpenoids occupy a large proportion. Among them, compounds **75** to **86** are pentacyclic triterpenes and compounds **8** to **9** are tetracyclic triterpenes, and their structures are shown in Figure 5.

### 4.5. Alkaloids

**Figure 6:**
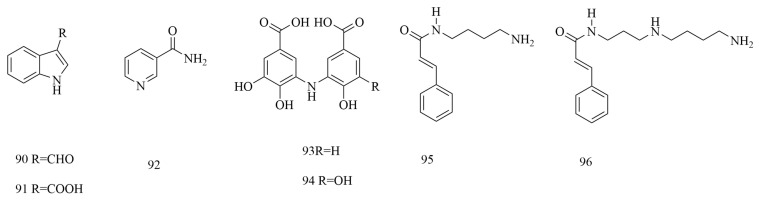
The structures of alkaloids in *V. coloratum*.

Alkaloids are non-primary metabolites that contain negative oxidized nitrogen atoms and exist in biological organisms. Alkaloids are rarely found in animals and are mainly distributed in the plant kingdom [54]. At present, 7 alkaloids (**90~96**) have been isolated and identified in *V. coloratum*, as shown in Figure 6. The alkaloids contained in *V. coloratum* have attracted much attention due to their significant antitumor activity, but this component also constitutes the main source of its toxic effects [55]. This unique efficacy–toxicity binary property presents a double challenge to clinical conversion, so it is worth paying attention to for future clinical applications.

### 4.6. Polysaccharides

Polysaccharides are polymerized by more than 10 monosaccharide molecules through glucoside bonds. For a long time in the past, polysaccharides were generally regarded as useless components. However, with the deepening of research, people gradually realized the important role of polysaccharides [56]. At present, three polysaccharides, VCP1, VCP2, and VCP3 from *V. coloratum* have been isolated, purified, and characterized in detail, according to the literature. The monosaccharide composition is determined by HPLC, and it is found that VCP1 is mainly composed of Glc and Gal, without GluA and GalA. VCP2 is composed of Ara with a high content of GalA and Gal and a small amount of Glc, Rha, and Man. VCP3 is mainly composed of Ara and GalA. There are high levels of Rha and Gal and small amounts of Glc and Man. The basic characteristics of VCP are shown in Table 2.

### 4.7. Lectins

Lectins are a class of non-immunogenic, non-enzymatic proteins derived from plants that can agglutinate cells and precipitate monosaccharide or polysaccharide complexes. Due to their specific binding affinity for saccharides or glycoconjugates, they play critical roles in multiple biological processes, including signal transduction, immune response, and plant defense mechanisms. Additionally, lectins exhibit cell-agglutinating activity through carbohydrate recognition, as well as antiviral, antifungal, and apoptosis- or autophagy-inducing activity [58]. At present, six kinds of lectins have been extracted, separated, and purified from *V. coloratum* in detail, namely CM-0, CM-1, CM-2, ACML-55, VCL, and ML. The relative molecular weights and sugar specificities of the corresponding subunits are shown in Table 3.

### 4.8. Other Compounds

**Figure 7:**
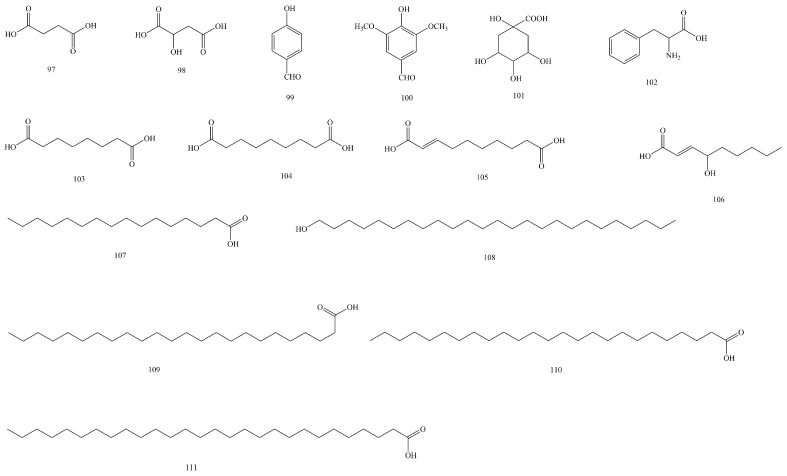
The structures of other compounds in *V. coloratum*.

In addition to the seven types of compounds mentioned above, fifteen other compounds were isolated and identified (**97–111**), including fatty acids such as lignoceric acid (**109**), cerotic acid (**110**), and octacosanoic acid (**111**); aliphatic dibasic acids such as nonanedioic acid (**104**); and amino acids such as phenylalanine (**106**). Their structures are shown in Figure 7.

## 5. Pharmacology

### 5.1. Anti-Inflammatory Effect

Rheumatoid arthritis (RA) is a chronic autoimmune disorder characterized by persistent synovial inflammation and progressive bone erosion, leading to structural joint deformities and functional impairment in affected individuals [61]. The etiology and pathogenesis of RA are complex. One study found that the extract of *V. coloratum* (EVC) had a certain effect on RA. The collagen-induced arthritis (CIA) model was successfully generated in DBA/1J mice through type II collagen immunization. The effect of EVC on rheumatoid arthritis was compared by toe thickness, joint index, and IgG level in mice. The mice were divided into the normal group, the model group, the methotrexate (MTX) group, and three groups treated with different doses of EVC (1, 2, 4 g/kg). After 28 days, an antibody-based cytokine microarray assay was employed to quantify the expression profiles of inflammation-associated cytokines and observe the toe condition of the mice. The study demonstrated a marked decrease in joint inflammation scores and digital swelling measurements, indicating that EVC effectively mitigates arthritic symptoms in collagen-induced arthritis (CIA) murine models while maintaining an excellent safety profile with no observed adverse effects [33]. Subsequently, researchers conducted pharmacological validation of the pharmacodynamic material basis based on metabolomics analysis. Twenty-two plasma-detectable flavonoid parent compounds and their metabolites act as the core therapeutic constituents of *V. coloratum* against rheumatoid arthritis. Specifically, components such as the demethylated viscumneoside VI derivative demonstrated significant anti-inflammatory efficacy. These findings provide mechanistic insights into impaired Phase II metabolism in RA pathophysiology and key data support for the metabolic mechanism of *V. coloratum* in the treatment of RA [62].

Inflammatory bowel disease (IBD) is a chronic immune-mediated disorder characterized by idiopathic inflammation of the gastrointestinal tract, predominantly affecting the ileum, the rectum, and the colon [63]. Clinical manifestations include diarrhea, abdominal pain, and even bloody stools. These diseases include ulcerative colitis (UC) and Crohn’s disease (CD) [64]. One study showed that the ethanol extract of *V. coloratum* (VCE) had a significant therapeutic effect on IBD. Mice were modeled with dextran sodium sulfate (DSS). In vivo studies were conducted on mice after daily oral administration of VCE (0–200 mg/kg), and the efficacy of VCE was determined by observing mouse body weight, disease activity index, and therapeutic index. The colonic tissue morphological changes of mice were observed with an endoscope, F4/80, zonula occludens-1 (ZO-1), matrix metalloprotease-2 (MMP-2), and MMP-9 were analyzed by histological analysis and immunoblot analysis, and the levels of serum IgE, IL-6, and TNF-*α* in the colonic tissue were detected. The results showed that the mice taking oral VCE had a reduced body weight, decreased intestinal epithelial tissue damage, intestinal bleeding, intestinal edema, and decreased levels of serum IgE, IL-6, and TNF-*α*. In an in vitro experiment, a colitis model co-cultured with RBL-2H3 and Caco-2 was constructed, and the effects of VCE on the activity of MMP-2/MMP-9 and the expression of ZO-1 were detected. The results showed that VCE inhibited the activation of Caco-2 cells by inflammatory mediators released by mast cells, reduced the expression of MMP-2/MMP-9, and restored ZO-1, which was consistent with the results of in vivo experiments. These results indicate that VCE has a good therapeutic effect on enteritis in mice. Therefore, VCE has a promising prospect for the future development of drugs for the treatment of IBD [65].

In another study, it was found that a partially purified extract of *V. coloratum* (PPE-SVC) and viscolin had therapeutic effects on neutrophil inflammation. In vitro experiments were conducted using human neutrophils activated by formyl-L-methionyl-L-leucyl-L-phenylalanine (FMLP) to detect the inhibitory effect of viscolin on O_2_^−^ generation and elastase release. The cAMP level was determined using enzyme-linked immunosorbent assay (ELISA), and the activities of phosphodiesterase (PDE) and adenylate cyclase (AC) were evaluated. The results showed that PPE-SVC effectively inhibited superoxide anion production and elastase release induced by FMLP, with IC_50_ values of 0.58 ± 0.03 μg/mL and 4.93 ± 0.54 μg/mL. Viscolin, a novel flavonoid derivative isolated from PPE-SVC, was also shown to inhibit superoxide anion production and elastase release and accelerate the re-release of cytoplasmic calcium in FMLP-activated human neutrophils. The inhibitory effect of viscolin is mediated by protein kinase A (PKA). PPE-SVC and viscolin inhibit the inflammatory response in human neutrophils by inhibiting PDE and thereby increasing cAMP [42]. This can be found in the study of its better anti-inflammatory effect.

A subsequent study showed that viscolin can reduce the expression of VCAM-1 in TNF-*α*-treated endothelial cells through the JNK/NF-*κ*B and ROS pathways, thus achieving anti-inflammatory effects. The researchers used Western blot analysis, RT-PCR to detect mRNA levels, luciferase reporter gene to detect promoter activity, ROS assay, and NADPH oxidase activity. The results showed that viscolin could significantly inhibit the expression of TNF-*α*-induced VCAM-1 protein and mRNA, decrease ROS production and NADPH oxidase activity, and reduce the adhesion of monocytes to endothelial cells, thus achieving anti-inflammatory effects. In subsequent animal experiments, the expression of VCAM-1 in the thoracic aorta of viscolin-pretreated mice was also significantly reduced under TNF-*α* stimulation, further verifying the results of the in vitro experiment. Viscolin demonstrates potent anti-inflammatory and antioxidant effects, suggesting its potential therapeutic applications in the prevention of atherosclerosis and management of inflammatory conditions [66].

Allergic asthma is a chronic condition that typically begins in early childhood. If left untreated, it may lead to life-threatening complications or persist throughout life [67]. A recent study found that *V. coloratum* has a certain effect on allergic asthma. The researchers studied the anti-inflammatory effects of PPE-SVC and its monomeric constituent viscolin on asthmatic models. BALB/c mice were sensitized with ovalbumin (OVA) and intraperitoneally injected with 5 mg/kg PPE-SVC and viscolin. The results demonstrated that both PPE-SVC and viscolin significantly attenuated airway hyperresponsiveness (AHR) in OVA-sensitized mice, which was manifested by a significant reduction in the Penh value. Both treatments significantly reduced the number of eosinophils in bronchoalveolar lavage fluid (BALF) and lung tissue. In the BALF, PPE-SVC and viscolin significantly reduced levels of the Th2-associated cytokine IL-5, but had no significant effects on IL-4 and IL-13. In addition, both groups decreased serum levels of OVA-specific IgE, but did not significantly alter Th2 cytokine secretion in spleen cell cultures. Therefore, PPE-SVC and viscolin can effectively alleviate asthma symptoms in OVA-sensitized mice by inhibiting the activation and migration of eosinophils and reducing the release of inflammatory mediators. The effect of PPE-SVC is better than that of viscolin, possibly because it contains other active ingredients [68]. This study provides an experimental basis for the application of *V. coloratum* in the treatment of allergic asthma.

### 5.2. Anticancer Effect

Global cancer epidemiology has undergone a marked upward trajectory in recent decades, with age-standardized incidence rates rising annually, potentially attributable to synergistic interactions between lifestyle modifications, environmental exposures, and demographic transitions characterized by extended longevity [69]. One study found that VCP has a certain effect on liver cancer. Using the isobaric tags for relative and absolute quantitation (ITRAQ) method, Chai et al. (2016) detected the inhibitory effect of VCP on HepG2 by using a Cell Counting Kit-8 (CCK-8) assay. VCP1, VCP2, and VCP3 of polysaccharides with different concentrations were used to study their inhibitory effects on HepG2 cells. The results showed that the inhibitory effect on HepG2 cells was gradually stronger with the increase in concentration. High concentrations of polysaccharides in the solution had an obvious inhibitory effect, while low concentrations of polysaccharides dissolved in the solution had no obvious inhibitory effect. Moreover, VCP2 had the strongest inhibitory effect on HepG2 cells. When the polysaccharide concentration reached 10.0 mg/mL, the proliferation inhibitory rate of VCP2 on HepG2 cells was the highest, with the inhibitory rate of 48%. VCP demonstrated significant antiproliferative activity against HepG2 cells in a dose-dependent manner [21].

On a global scale, lung cancer is still the most common cause of cancer deaths [70]. It has been found that 1,7-bis(4-hydroxyphenyl)-1,4-heptadien-3-one (EB30), a compound extracted from *V. coloratum*, can induce apoptosis of lung cancer cells through the PI3K/AKT and ERK1/2 pathways, so as to achieve a therapeutic effect on lung cancer. Fan et al. (2018) screened a *V. coloratum* extract using the CCK-8 assay, and the screening results showed that EB30 could inhibit the proliferation of lung cancer cells. The survival rate of A549 cells and NCI-H292 cells decreased in a dose-dependent manner when the EB30 concentration was 10–40 μM (the higher the dose, the lower the survival rate), and the IC_50_ value of the EB30 treatment for 48 h was 8.61 μM and 12.71 μM. At the same time, in order to verify the specific cytotoxicity of EB30 on tumor cells, HBE cells were treated with EB30 for 48 h. The results showed that the IC_50_ value of EB30 was 28.58 µm, and its toxicity was 2.25 times and 3.32 times lower than that of A549 cells and NCI-H292 cells, indicating that EB30 was selective for lung cancer cells [71].

In addition, a subsequent study found that EB30 has a great effect on breast cancer. The in vitro cytotoxic activity of EB30 against 12 cancer cell lines was determined using a methylthiazolyldiphenyl tetrazolium bromide (MTT) cell proliferation assay. EB30 was compared with cisplatin (CDDP) and tested against twelve types of cancer cells. The results demonstrated that EB30 exhibited cytotoxic activity against 12 cancer cell lines at micromolar concentrations. Notably, EB30 showed a significantly higher efficacy than CDDP against four breast cancer cell lines (SKBR3, MDA-MB-231, MCF-7, and MDA-MB-453) and the SKMEL-28 melanoma cell line. The IC_50_ values of EB30 in these five cell lines were markedly lower than those of CDDP. On colon cancer cells (HT-29) and lung cancer cells (CALU-3), there was no significant difference in IC_50_ values between the EB30 and CDDP groups. The IC_50_ of other tumor cells treated with EB30 was significantly higher than with CDDP, but less than 11 mmol/L. In addition, EB30 was more potent against four human breast cancer cell lines compared to the positive control CDDP and showed a dose-dependent effect. Subsequent experiments on EB30 with normal human cells showed that EB30 had a small inhibitory effect on normal cells, and the inhibition rate of each group was less than 5%, which is much less than the inhibition rate of EB30 at the same concentration for cancer cells [72]. This suggests that EB30 is selective in its toxic effects on cancer cells and normal cells. In the follow-up, we were able to conduct in-depth studies on EB30, and the findings demonstrate that EB30 exhibits promising therapeutic potential for the development of lung and breast cancer treatments.

Human osteosarcoma is a major malignancy affecting the bones of children and young adults aged 19 to 29, as well as adults over 60 years old [73]. Studies have shown that the antitumor effects of *V. coloratum* alkaloids on human osteosarcoma cells (U2OS) and their mechanisms have been evaluated through in vitro experiments [74]. U2OS cells were treated with different concentrations of *V. coloratum* alkaloids and chemotherapy drug 5-fluorouracil (5-FU). The IC_50_ value of *V. coloratum* was 7 μg/mL (CCK-8 method), and the efficacy of *V. coloratum* alkaloids was compared with that of 5-FU. The results showed that the inhibitory effect of *V. coloratum* on the proliferation of U2OS cells was significantly stronger than that of 5-FU. Terminal deoxynucleotidyl transferase-mediated deoxyuridine triphosphate nick end labeling staining and immunocytochemical staining of caspase 3 detection confirmed that *V. coloratum* can significantly increase the apoptosis rate of U2OS cells, and the effect is better than that of 5-FU. A Transwell migration experiment further showed that the invasion ability of U2OS cells treated with a *V. coloratum* extract was lower than that in the 5-FU treatment group. Comprehensive experimental data showed that *V. coloratum* can not only significantly inhibit the proliferative activity of osteosarcoma cells in vitro, but also play a multi-target antitumor role by inducing cell apoptosis and inhibiting invasion and migration, which is better than the traditional chemotherapy drug 5-FU.

In addition, other studies also confirmed that *V. coloratum* alkaloids have good anticancer effects. Peng et al. (2005) studied the anticancer effects of *V. coloratum* alkaloids through in vitro and in vivo experiments [75]. The MTT method was used in in vitro experiments to determine the inhibitory effects of *V. coloratum* alkaloids on the growth of esophageal cancer cell line Eca109 cells and human breast cancer MCF-7 cells. The results showed that *V. coloratum* had a significant inhibitory effect on Eca109 cells and MCF-7 cells in a dose-dependent manner. When the dose of *V. coloratum* alkaloids was 120 mg/kg, the inhibitory rates of Eca109 cells and MCF-7 cells were 67.05% and 70.23%. In an in vivo experiment, hepatoma H22 suspension was inoculated into the subcutaneous area of the right forelimb of mice at 0.2 mL/mouse. Twenty-four hours later, the inoculated mice were randomly divided into 5 groups with 10 mice in each group, including the control group, the 5-FU group, and the high-, medium-, and small-dosage groups (120, 90, and 60 mg/kg). Mice in the *V. coloratum* group were administered treatment once a day by intragastric administration. The 5-FU group was administered an intraperitoneal injection (0.2 mL) once a day. The control group was given 1% CMC-Na solution every day. All groups underwent continuous administration for 7 days. The mice were killed 1~3 days after drug withdrawal, and the tumor blocks were removed and weighed. The tumor inhibition rate was calculated. The results showed that *V. coloratum* alkaloids could inhibit tumor growth and prolong the survival time of tumor-bearing animals. In a dose-dependent manner, the inhibitory rate of *V. coloratum* alkaloids on tumor reached 72.87% when the dose of *V. coloratum* alkaloids was 120 mg/kg, which was higher than the inhibitory rate of 70.54% in the control group (5-FU).

### 5.3. Antioxidant Effect

Oxidative stress refers to a state of imbalance between oxidation and antioxidants in the body [76]. The incidence of chronic diseases such as diabetes, hypertension, and Alzheimer’s disease is gradually increasing worldwide [77]. The causes of these diseases are related to oxidative stress. Mistletonone (**69**) is a chalcone isolated and identified from *V. coloratum*. Researchers used the ESR technology to detect its antioxidant capacity, and the results showed that mistletonone has a scavenging effect on hydroxyl radicals and superoxide anion radicals. Its IC_50_ values are 0.485 mM and 0.273 mM. These findings demonstrate that mistletonone exhibits potent antioxidant activity [17]. In addition, it was found that VCP has a significant antioxidant effect. The scavenging ability of DPPH and hydroxyl radicals was tested by means of an in vitro antioxidant experiment. The results demonstrated that the DPPH radical scavenging rate of VCP increased from 36.36% ± 3.43% to 80.01% ± 2.31% at concentrations ranging from 2 to 6 mg/mL. The EC_50_ value of VCP was determined to be 3.02 mg/mL. However, no significant dose-dependent enhancement in DPPH radical scavenging activity was observed beyond this concentration of 6 mg/mL. At the concentration of 2–10 mg/mL, the hydroxyl radical scavenging activity of VCP increased from 7.27% ± 1.21% to 38.26% ± 1.79%. These data show that VCP has a significant antioxidant capacity [78].

Yao et al. (2006) extracted *V. coloratum* with ethanol and isolated and identified 5 compounds, namely compounds **6**, **13**, **14**, **15**, and **16**. These five compounds were evaluated for their antioxidant capacity on the scavenging of hydroxyl radicals and superoxide anion radicals. The effective activity of each sample was expressed as IC_50_. The results showed that their IC_50_ values for hydroxyl radical scavenging were 0.25, 0.21, 0.18, 0.28, and 0.33 mM. For the superoxide anion radical scavenging, the IC_50_ values were 0.23, 0.39, 0.25, 0.30, and 0.49 mM. Therefore, the five isolated compounds showed significant scavenging effects on hydroxyl radicals and superoxide anion radicals [35]. Through these studies, it can be found that *V. coloratum* has great potential in antioxidant activity, and these findings provide a theoretical basis for the targeted creation of new natural antioxidants and an innovative candidate substance group for the upgrading of antioxidant products in food, medicine, and other fields.

### 5.4. Anti-Cardiovascular Disease Effect

Cardiovascular diseases remain a leading global cause of morbidity and mortality, a trend exacerbated by the escalating prevalence of modifiable risk factors, including obesity, tobacco use, chronic stress, hypercholesterolemia, diabetes mellitus, hypertension, physical inactivity, and suboptimal nutritional intake [79]. One study found that *V. coloratum* flavonoids (VCF) have a certain protective effect on ischemic myocardial injury in the body, and the fast response action potential (PAF) has a regulatory role [80]. Wistar rats were divided into five groups: control group, ischemia (MI) group, ischemia low-VCF group (MI-LV), ischemia high-VCF group (MI-LH), and BN52021 (PAF antagonist) group (10 mg/kg). The animal model was represented by rats with left anterior descending branch coronary artery ligation. Muscle cells were isolated and their Ca^2+^ concentration was measured. The results showed that 10 mg/kg BN52021 as a control had a small effect on the size of the infarct area in rats with a low concentration of VCF and a larger effect on the size of the infarct area in rats with a high concentration of VCF. Determination of the Ca^2+^ concentration showed that VCF protected cardiomyocytes by inhibiting PAF-induced intracellular calcium overload. VCF may therefore be used to develop new drug PAF blockers to improve heart function and prevent heart damage during ischemia or reperfusion.

Arrhythmias, which are the origin and/or conduction disorders of heart activity leading to abnormal frequency and/or rhythm of heartbeats, are an important group of cardiovascular diseases [81]. Studies have shown that VCF has a certain therapeutic effect on tachyarrhythmia, but not on chronic arrhythmia. Wu et al. (1994) used the glass microelectrode technique to detect the influence of PAF on dog Purkinje cells and guinea pig ventricular papillary muscle cells and conducted a preliminary analysis of the influence of VCF on the transmembrane ion flow of each phase of PAF through selective membrane channel blockers [82]. The results showed that PAF changes were observed in the VCF-treated dog Purkinje cells and guinea pig ventricular papillary muscle cells, and the changes tended to be stable after 10 min. There were no significant changes in Vmax and rapid repolarization 1-phase amplitude (P1A) of PAF 10 min after treatment. The maximum diastolic potential (MDP) and the action potential amplitude (APA) were slightly reduced. The duration of action potential (APD) and the duration of recombination to 50% action potential duration (APD50) and APD90 were significantly shortened. Although ERP was shortened compared to before medication, the ratio of ∆ERP/∆APD was significantly increased after medication, so ERP was relatively prolonged. The results showed that VCF was effective for tachyarrhythmia, and the electrophysiological effect of PAF appeared at 2 min, tended to be relatively stable at 10 min, and the effect of drug washout disappeared at 15 min. It can be observed that VCF acted quickly, maintained a short time, and was reversible.

In previous studies, viscolin was found to exhibit anti-inflammatory effects, and subsequent research further demonstrated its therapeutic potential in preventing and treating vascular proliferative diseases [42,66]. Chen et al. (2016) divided C57BL/6J mice into two treatment groups: (1) the control group with 50 μL PBS and (2) the experimental group with 100 μg/kg body weight of viscolin in 50 μL of PBS; intracavitary mechanical injury was used for modeling. After half a month of mechanical damage, the mice were treated with drugs. Since vascular SMC proliferation and migration were induced by PDGF-BB, the influence of viscolin on the proliferation and migration of PDGF-BB-treated HASMCs was tested using the MTT, BrdU incorporation, and wound healing assays. The results showed that viscolin at 30–40 μM significantly inhibited the proliferation and migration of HASMCs induced by PDGF-BB. To examine the effect of viscolin on endothelial cell growth, a crystal violet cell proliferation assay was performed. The results showed that the cell growth of HUVECs treated with viscolin was 1.00 ± 0.01, 1.05 ± 0.02, 1.02 ± 0.01, 0.98 ± 0.01, and 1.01 ± 0.02, indicating that viscolin had no inhibitory effect on the growth of endothelial cells. It was found that viscolin inhibited the proliferation and migration of HASMCs induced by PDGF-BB and had no effect on endothelial cell growth [83]. Therefore, viscolin has great potential for the prevention and treatment of vascular proliferative diseases.

### 5.5. Other Effects

Hepatitis B virus (HBV) infection is a major public health problem around the world [84]. It is an infectious disease mainly caused by the hepatitis B virus. The main clinical manifestations are anorexia, nausea, upper abdominal discomfort, hepatic pain, and fatigue [85]. Recently, a study found that VCP1, VCP2, and VCP3 have obvious inhibitory effects on HBV-DNA replication. A CCK-8 kit was used to detect the proliferation of HepG2.2.15 cells, and the results showed that *V. coloratum* had a certain inhibitory effect on the proliferation of HepG2.2.15 cells. PQ-PCR was used to detect the effects of VCP on HBV-DNA. The results showed that VCP significantly inhibited the replication of HBV-DNA, and the maximum inhibitory rate was 28.192% ± 0.021%; the inhibitory effect on HBsAg and HBeAg secretion was the highest, 5.676% ± 0.012% and 4.880% ± 0.010%, when the concentration was 10 mg/mL. The above three experimental results were concentration-dependent, indicating that VCP may be a great antiviral agent [78].

Another study found that the ethyl acetate extract of *V. coloratum* has a significant effect on inhibiting osteoporosis. After solvent fractionation (hexane, ethyl acetate, n-butanol, water), the inhibitory ability of each component on osteoclast formation and bone absorption was evaluated by means of in vitro and in vivo experiments. The results showed that the ethyl acetate component (EtOAc) significantly inhibited osteoclast-like multinucleate cell formation at a low concentration of 20 μg/mL, and the inhibition rate was 94.9%. It also effectively inhibited bone resorption induced by parathyroid hormone (PTH) and reduced 45Ca release. In vivo, oral administration of the EtOAc component (50/100 mg/kg) for 6 weeks in an ovariectomized mouse model (simulating postmenopausal osteoporosis) significantly improved bone parameters and inhibited the decrease in cancellous bone BMD caused by ovariectomy. The BMD of ovariectomized rats in the EtOAc-100 group was 203 ± 19 mg/cm^3^, in the EtOAc-50 group—151 ± 27 mg/cm^3^, and in the 17*β*-estradiol group—198 ± 51 mg/cm^3^. The effect of oral EtOAc was superior to that of 17*β*-estradiol in terms of bone strength and improved compression strength and cortical bone thickness without the adverse effects of uterine weight gain [15]. This study was the first to show that the ethyl acetate component of *V. coloratum* improves bone metabolism by inhibiting osteoclast activity without estrogen-related side effects.

**Figure 8:**
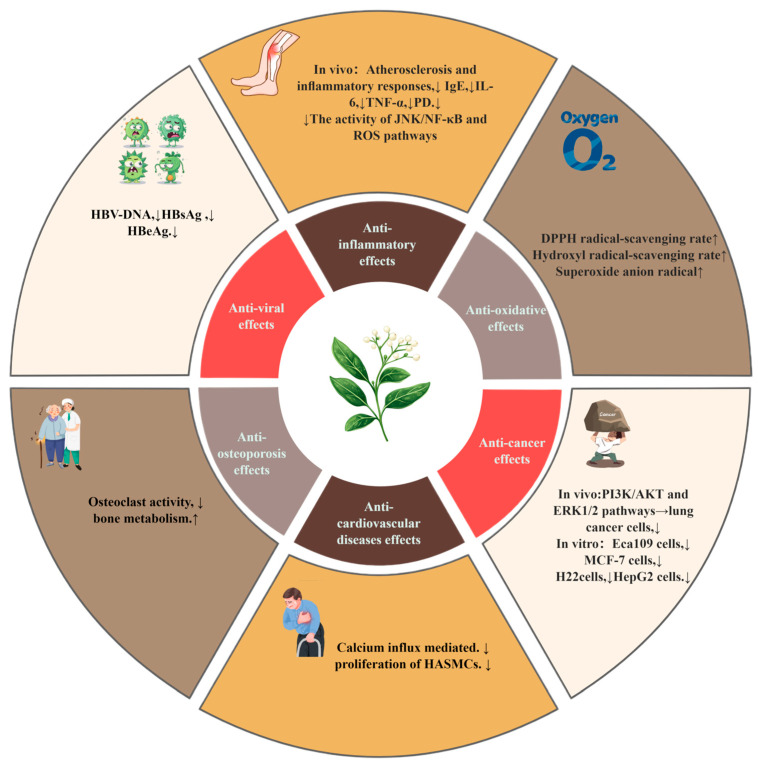
The effects of *V. coloratum*: ↑: improved or promoted; ↓: inhibited or reduced.

*V. coloratum* has multiple pharmacological effects, such as the anti-inflammatory effect, the anticancer effect, the antioxidant effect, and other effects. The overview of its pharmacological effects is shown in Table 4 and Figure 8.

## 6. Pharmacokinetics

The pharmacokinetic analysis of four components in mice was conducted using an HPLC system [86]. The four components were compounds **6** (Hedt-III), **29** (Hedt-II), **32** (Hedt-I), and **47** (Syri). After an intravenous injection of three different formulations, a monomer solution (MONO), a mixture solution (MIX), and *V. coloratum* extracts (VCEs), the pharmacokinetic interactions of the co-existing components of *V. coloratum* were analyzed. The results showed that in the Syri group, t_1/2_ was significantly longer in the VCE and MIX groups than in the MONO group, but there was no significant difference in the AUC, suggesting that other components may inhibit its metabolism. In the Hedt-I group, there was no significant difference in pharmacokinetic parameters among the three groups, indicating that other components had no significant effect on its metabolism. In the Hedt-II group, t_1/2_ did not differ significantly between the three groups, and the AUC was significantly lower in the VCE and MIX groups than in the MONO group, suggesting that co-existing components may accelerate their metabolism. In the Hedt-III group, the AUC in the VCE group was significantly higher than in the MIX and MONO groups, and t_1/2_ was significantly longer in the VCE and MIX groups than in the MONO group, indicating that other components in the extract may inhibit its metabolism. From the above conclusions, we observe that there are complex pharmacokinetic interactions among the components of *V. coloratum* involving the interaction of absorption, metabolism, and elimination.

The UHPLC–MS/MS method was used to quantitatively determine nine *V. coloratum* flavonoid extracts, compound **5** (Hedt-IV), compound **6** (Hedt-III), compound 17 (Httf), compound **22** (Isor), compound **26** (Rham-I) compound **29** (Hedt-II), compound **30** (Rham-II), compound **32** (Hedt-I), and compound **37** (Rham-III), in a rat pharmacokinetic study [87]. The results showed that the t_1/2_ (h) values of the nine compounds in rats were 0.67 ± 0.33, 3.32 ± 1.14, 0.49 ± 0.22, 0.81 ± 0.22, 1.24 ± 0.35, 1.12 ± 0.42, 0.74 ± 0.17, 2.12 ± 1.36, and 0.81 ± 0.22. Through these data, it can be found that the half-lives of the other eight compounds, except Hedt-III, are shorter, less than 3 h, which may be related to their larger molecular structures. This study provided an efficient analytical tool for the pharmacokinetic study of the multi-component *V. coloratum*, revealed the metabolic differences of different structural components, and laid a foundation for clinical dose optimization and mechanism research. The advantages of UHPLC–MS/MS in the analysis of complex components of the traditional Chinese medicine were also emphasized.

The distribution of Hedt-III in plasma and tissues of mice was studied in more detail using the HPLC method [88]. After 13.2 mg·kg^−1^ of Hedt-III was injected intravenously in mice, t_1/2_, *α* and t_1/2_, *β* values were 0.06 ± 0.01 h and 1.27 ± 0.31 h, AUC and CLtot values were 16.04 ± 3.19 mg·kg^−1^·h^−1^ and 0.85 ± 0.17 mg·kg-1·h^−1^, and their distribution characteristics showed rapid drug elimination. It is mainly distributed in the liver and the small intestine, and low concentrations could still be detected in blood after 5 h. The tissue distribution data showed that the concentration of Hedt-III was higher in the liver and the small intestine, but it was not detected in brain tissue 0.083 h^-1^ h after administration. Therefore, its metabolic and distribution characteristics in rats can be clarified, providing key data support for future translational medicine research and formulation optimization.

DHDK is a diphenylheptane compound isolated from *V. coloratum*. DHDK showed great antitumor activity. The researchers analyzed the plasma concentration and tissue distribution of DHDK by means of LC-ESI-MS/MS and evaluated its metabolic characteristics [72]. The results showed that DHDK had a fast metabolism (t_1/2_ = 0.064 h), a small distribution volume, and was mainly concentrated in lung tissue, which revealed its potential in the treatment of lung cancer. Compared with the pharmacokinetic parameters of cisplatin, it was found that the renal clearance rate was lower than that of cisplatin, therefore, DHDK had lower renal toxicity.

## 7. Toxicology

Researchers found that some *Viscum* spp. plants are toxic [89] and that the lectins, alkaloids, and other components of *V. coloratum* are toxic sources. Studies on the toxicity of *V. coloratum*: acute toxicity tests were conducted with aqueous extracts (AQs) [90], petroleum ether extracts (PEs), and crude ethanol extracts (CEEs) of *V. coloratum* in mice via intragastric administration. The LD_50_ value was used as the core index to evaluate the toxicity characteristics of different extracts. The results showed that the LD_50_ values of the CEE group and the AQ group were 7.67 g/kg and 0.65 g/kg, and no dead mice were found in the PE group. The CEE group showed significantly higher values than the other groups, indicating relatively low acute toxicity, while the AQ group showed an extremely strong acute toxicity. Follow-up studies could therefore be combined with subacute toxicity tests by repeating doses for 28 days and genotoxicity tests to fully assess the toxicological profile and ensure the safety of future clinical drug use.

At present, systematic studies on the toxicology of *V. coloratum* are relatively scarce, while the toxic effects of its close relative *V. album* L. have been thoroughly investigated [91]. This difference in research may be due to the influence of traditional medicine. In the traditional Chinese medicine, the clinical application of *V. coloratum* mainly relies on its dried stems and branches as the medicinal site, while other tissues, such as fruit, are usually excluded from the medicinal field. Due to the long-term focus on site-specific pharmacodynamic development, research into the underlying toxicological mechanisms has been significantly underinvested, such as into the distribution of toxic components across the plant or dose-dependent risk. In contrast, toxicity studies of *V. album* L. reveal its toxicity characteristics more comprehensively [92]. The limitation of this research direction suggests that the toxicity profile of different tissues, including non-medicinal parts of *V. coloratum*, should be systematically evaluated with modern toxicological methods in the future to improve its safety evaluation system.

## 8. Conclusions and Prospects

In recent years, *V. coloratum* has attracted much attention due to its extensive biological activities. The botanical characteristics, traditional applications, phytochemistry, and pharmacological effects of this species were reviewed, and the research progress in toxicology is discussed in this paper. Based on previous studies, systematic characterization of 111 compounds was summarized, including flavonoids, terpenoids, phenylpropanoids, etc. Pharmacological studies confirmed that *V. coloratum* plays a regulatory role in tumor regulation, inhibition of inflammatory factors, intervention of the viral replication cycle, oxidative stress balance, and other key pathological links. Therefore, *V. coloratum* has demonstrated significant clinical translational potential in such areas as synergistic cancer therapy, targeted anti-inflammatory treatment, and the regulation of oxidative stress. Its multi-component synergistic mechanism offers a crucial foundation for the development of novel plant-based therapeutic strategies. In short, as a plant resource with important medicinal value, *V. coloratum* has attracted much attention from the academic community for its rich phytochemical components and multi-pharmacological properties.

Although a variety of bioactive substances have been isolated and identified from *V. coloratum*, there are still many key problems in related research. For example, there is significant interspecific morphological convergence and genetic diversity overlap among *Viscum* plants, resulting in a high misjudgment rate of traditional taxonomic methods. In the future, we can introduce a multi-omics integration identification system. For example, ITS2 and psbA-trnH double DNA barcodes have been used to construct germplasm-specific molecular tags, and species-specific metabolic markers have been screened by LC–MS non-targeted metabolomics to reduce the error rate. In addition, there are significant limitations in the current quality control model of ChP, 2020, that relies on syringin’s single index (≥0.040%). In the future, we can try to use network pharmacology to target multi-target component groups that simultaneously regulate the PI3K/Akt, MAPK, and Nrf2 pathways, such as the combination of viscumamide III and quercetin-3-O-glucoside, and establish a comprehensive scoring system based on the effect component index. The existing clinical studies need to expand the sample size and improve the experimental design to confirm the safety boundary and therapeutic efficacy of clinical application. A considerable proportion of the discovered compounds have not been systematically characterized, and their structure–activity relationships and molecular mechanisms need to be further analyzed. As a hemiparasitic plant, *V. coloratum* parasitizes on diverse host trees. Currently, comprehensive chemical profiling data—both qualitative and quantitative—for *V. coloratum* growing on different host species are lacking. Future studies should employ UPLC-QTOF-MS/MS to characterize its components, establish a spectral library, and quantitatively correlate chemical profiles with the host tree species. This approach will provide a foundation for quality control and biosynthetic pathway studies of *V. coloratum*. The purpose of this review is to establish a systematic knowledge framework for *V. coloratum* research, so that future studies can elucidate the mechanism of action of active ingredients through multi-omics techniques, establish accurate structure–activity relationship models using computational chemistry, and develop novel drugs based on the mechanism of action. These breakthroughs in research direction can not only deepen the scientific understanding of the traditional efficacy of *V. coloratum*, but also provide a molecular basis and technical support for the development of innovative drugs. It is expected that this review can provide a theoretical basis for the in-depth development and utilization of *V. coloratum* and promote the transformation process of traditional medicinal plants into modern therapeutic drugs.

## Figures and Tables

**Table 1:** Compounds isolated from *V. coloratum*.

No	Compounds	Molecular Formula	Extraction Solvent	Parts of the Plant	References
Flavonoids					
**1**	Sakuranetin	C_16_H_14_O_5_	Water	Branches and leaves	[33]
**2**	Naringenin	C_15_H_12_O_5_	Water	Branches and leaves	[33]
**3**	Eriodictyol	C_15_H_12_O_6_	50% (*v*/*v*) methanol–water	Aboveground parts	[16]
**4**	Hesperetin	C_16_H_14_O_6_	Water	Branches and leaves	[33]
**5**	Homoeriodictyol	C_16_H_14_O_6_	Water	Branches and leaves	[34]
**6**	Homoeriodictyol-7-O-*β*-D-glucoside	C_22_H_24_O_11_	95% ethanol	Stems and leaves	[34,35]
**7**	Chrysin	C_15_H_10_O_4_	50% (*v*/*v*) methanol–water	Aboveground parts	[16]
**8**	7,3′,4′-Trimthylquercetin	C_18_H_16_O_7_	75% ethanol	Branches and leaves	[36]
**9**	Quercetin	C_15_H_10_O_7_	Water	Branches and leaves	[33]
**10**	Isorhamnetin	C_16_H_12_O_7_	Water	Branches and leaves	[33]
**11**	Quercetin-3,3′-dimethyl ether	C_17_H_14_O_7_	95% ethanol	Stems and leaves	[37]
**12**	Eupatorin	C_18_H_16_O_7_	Water	Branches and leaves	[33]
**13**	(2S)-homoeriodictyol-7,4′-di-O-*β*-D-glucopyranoside	C_28_H_34_O_16_	90% ethanol	Branches and leaves	[35]
**14**	(2R)-eriodictyol 7,4′-di-O-*β*-D-glucopyranoside	C_27_H_32_O_16_	90% ethanol	Branches and leaves	[35]
**15**	(2S)-eriodictyol-7-O-*β*-D-glucopyranoside	C_21_H_22_O_11_	90% ethanol	Branches and leaves	[35]
**16**	(2S)-naringenin-7-O-*β*-D-glucopyranoside	C_21_H_22_O_10_	90% ethanol	Branches and leaves	[35]
**17**	5-Hydroxy-3,7,3′-trimethoxyflavone-4′-O-*β*-D-glucoside	C_24_H_26_O_12_	50% methanol	Aboveground parts	[16,34,37]
**18**	Pachypodol	C_18_H_16_O_7_	50% methanol	Aboveground parts	[37]
**19**	Ombuine	C_17_H_14_O_7_	95% ethanol	Stems and leaves	[37]
**20**	Hyperoside	C_21_H_20_O_12_	50% (*v*/*v*) methanol–water	Aboveground parts	[16]
**21**	Rham-I	C_23_H_24_O_12_	50% methanol	Aboveground parts	[34]
**22**	Isorhamnetin-3-O-*β*-D-glucoside	C_22_H_22_O_12_	50% methanol	Aboveground parts	[34]
**23**	5,7,4′-Trihydroxy-3,3′-dimethoxyflavone	C_17_H_14_O_7_	50% methanol	Aboveground parts	[34]
**24**	Rhamnazine	C_17_H_14_O_7_	95% ethanol	Stems and leaves	[38]
**25**	Rhamnazin-3-O-*β*-D-glucoside	C_23_H_24_O_12_	95% ethanol	Stems and leaves	[37]
**26**	Viscumneoside I	C_27_H_32_O_15_	Water	Stems and leaves	[15]
**27**	Viscumneoside II	C_25_H_26_O_12_	95% ethanol	Stems and leaves	[39]
**28**	Viscumneoside III	C_27_H_32_O_15_	50% methanol	Aboveground parts	[37]
**29**	Viscumneoside IV	C_29_H_32_O_16_	95% ethanol	Stems and leaves	[40]
**30**	Viscumneoside V	C_32_H_40_O_19_	Water	Branches and leaves	[33]
**31**	Viscumneoside VI	C_24_H_26_O_12_	95% ethanol	Stems and leaves	[37]
**32**	Viscumneoside VII	C_34_H_40_O_20_	Water	Branches and leaves	[33]
**33**	Viscumneoside VIII	C_40_H_48_O_24_	95% ethanol	Stems and leaves	[41]
**34**	viscumneoside IX	C_28_H_32_O_16_	95% ethanol	Stems and leaves	[41]
**35**	Viscolin	C_19_H_24_O_6_	Methanol	Stems	[42]
**36**	Rham-III	C_35_H_42_O_21_	50% (*v*/*v*) methanol–water	Aboveground parts	[16]
**37**	Flavoyadorinin-B	C_23_H_24_O_11_	50% (*v*/*v*) methanol–water	Aboveground parts	[16]
**38**	Eriocitrin	C_27_H_32_O_15_	Water	Branches and leaves	[33]
Phenylpropanoids					
**39**	Coumarin	C_9_H_6_O_2_	50% (*v*/*v*) methanol–water	Aboveground parts	[16]
**40**	Cinnamic acid	C_9_H_8_O_2_	50% (*v*/*v*) methanol–water	Aboveground parts	[16]
**41**	Caffeic acid	C_9_H_8_O_4_	95% ethanol	Aboveground parts	[43]
**42**	Ferulic acid	C_10_H_10_O_4_	95% ethanol	Aboveground parts	[43]
**43**	Curcumene A	C_15_H_22_O_2_	95% ethanol	Stems and leaves	[44]
**44**	Chlorogenic acid	C_16_H_18_O_9_	Water	Branches and leaves	[33]
**45**	Liquidamboside	C_24_H_22_O_10_	50% (*v*/*v*) methanol–water	Aboveground parts	[16]
**46**	Syringin	C_17_H_24_O_9_	Water	Branches and leaves	[16,33,37]
**47**	5,6-Dehydro-4″-de-O-methylcentrolobin	C_19_H_20_O_3_	95% ethanol	Stems and leaves	[44]
**48**	(2R,3S,4S,6S)-6-(4-hydroxyphenethyl)-2-(4-hydroxyphenyl)-tetrahydro-2H-pyran-3,4-diol	C_19_H_22_O_5_	95% ethanol	Stems and branches	[45]
**49**	(1R,2S,3S,5S)-2,3-dihydroxy-3′,3″-dimethoxy-4′de-O-methylcentrolobine.	C_21_H_26_O_7_	95% ethanol	Stems and branches	[45]
**50**	(+)-Lariciresinol-9-O-*β*-D-glucopyranoside	C_26_H_34_O_11_	95% ethanol	Stems and leaves	[44]
**51**	Aketrilignoside B	C_28_H_36_O_14_	95% ethanol	Stems and leaves	[44]
**52**	Alangilignoside C	C_28_H_38_O_13_	95% ethanol	Stems and leaves	[44]
**53**	(+)-Isolariciresinol-9′-O-*β*-glucopyranoside	C_26_H_34_O_11_	95% ethanol	Stems and leaves	[44]
**54**	(6R,7S,8S)-7*α*-[(*β*-D-glucopyranosyl)-oxy]-1-methoxyisolariciresinol	C_27_H_36_O_12_	95% ethanol	Stems and leaves	[44]
**55**	(8R,7′S,8′S)-7*α*-[(*β*-D-glucopyranosyl)-oxy]- lyoniresinol	C_28_H_38_O_13_	95% ethanol	Stems and leaves	[44]
**56**	Zhebeiresinol	C_14_H_16_O_6_	75% ethanol	Stems and leaves	[46]
**57**	(+)-Epipinoresinol	C_20_H_22_O_6_	75% ethanol	Branches and leaves	[45]
**58**	Syringaresinol	C_22_H_26_O_8_	50% (*v*/*v*) methanol–water	Aboveground parts	[16]
**59**	Syringaresinol-O-*β*-D-glucopyranoside	C_29_H_40_O_13_	95% ethanol	Stems and leaves	[15]
**60**	pinoresinol-4-O-*β*-D-apiosly-(1→2)-*β*-D-glucoside	C_31_H_40_O_15_	95% ethanol	Aboveground parts	[44]
Diphenylheptanes					
**61**	1,7-Bis(4-hydroxyphenyl)-heptane-3,5-diol	C_19_H_24_O_4_	95% ethanol	Stems and branches	[45]
**62**	(3R,5R)-3,5-dihydroxy-1-(3,4-dihydroxyphe-nyl)-7-(4-hydroxyphenyl)-heptane	C_19_H_24_O_5_	95% ethanol	Stems and leaves	[44]
**63**	(3S,5S)-1-(4-hydroxy-3-methoxyphenyl)-7-(4-hydroxyphenyl)-heptane-3,5-diol	C_20_H_26_O_5_	95% ethanol	Stems and leaves	[44]
**64**	Diphenylheptane C.	C_23_H_32_O_8_	95% ethanol	Stems and leaves	[44]
**65**	1,7-Bis(4-hydroxyphenyl)-1,4-heptadien-3-one	C_19_H_18_O_3_	95% ethanol	Stems and leaves	[37]
**66**	1,7-Di-(4-hydroxyphenyl)-4E,6E-heptadiene-3-ketone	C_19_H_18_O_3_	95% ethanol	Stems and leaves	[44]
**67**	Diphenylheptane B	C_19_H_22_O_4_	95% ethanol	Stems and leaves	[44]
**68**	Mistletonone	C_19_H_20_O_5_	90% ethanol	Branches and leaves	[17]
**69**	1,7-Bis(4-hydroxyphenyl)-5-methoxyhept-1-en-3-one	C_20_H_22_O_4_	95% ethanol	Stems and branches	[45]
Terpenoids					
**70**	Loliolide	C_11_H_16_O_3_	75% ethanol	Branches and leaves	[36]
**71**	(1R,7S)-1,12,13-trihydroxybisabola-3,10-diene.	C_15_H_26_O_3_	95% ethanol	Stems and branches	[45]
**72**	(2Z,4E)-5-((S)-1-hydroxy-2,6,6-trimethyl-4-oxocyclohex-2-en-1-yl)-3-methylpenta-2,4-dienoic acid	C_15_H_20_O_4_	95% ethanol	Stems and branches	[45]
**73**	Vomifoliol	C_13_H_20_O_3_	95% ethanol	Stems and leaves	[44]
**74**	Eriobotroside II	C_24_H_38_O_11_	95% ethanol	Stems and leaves	[44]
**75**	*β*-Amyrin	C_30_H_50_O	75% ethanol	Branches and leaves	[36]
**76**	Erythordiol	C_30_H_50_O_2_	95% ethanol	Aboveground parts	[46]
**77**	Oleanolic acid	C_30_H_48_O_3_	Water	Branches and leaves	[16,37]
**78**	Alstolarnoid D	C_32_H_52_O_4_	95% ethanol	Stems and leaves	[44]
**79**	Maslinic acid	C_30_H_48_O_4_	70% ethanol	Aboveground parts	[33]
**80**	Oleanane-type triterpene	C_30_H_48_O_4_	95% ethanol	Stems and leaves	[44]
**81**	*β*-Acetylamyrin	C_32_H_52_O_2_	95% ethanol	Stems and leaves	[47]
**82**	*β*-Amyrin acetate	C_32_H_52_O_2_	75% ethanol	Branches and leaves	[36]
**83**	Lupeol acetate	C_32_H_52_O_2_	95% ethanol	Stems and leaves	[37]
**84**	Betulonic acid	C_30_H_46_O_3_	50% (*v*/*v*) methanol–water	Aboveground parts	[16]
**85**	Lupeol	C_30_H_50_O	75% ethanol	Branches and leaves	[36]
**86**	3-Epi-betulinic acid	C_30_H_48_O_3_	75% ethanol	Branches and leaves	[36]
**87**	*β*-Sitosterol	C_29_H_50_O	50% (*v*/*v*) methanol–water	Aboveground parts	[37]
**88**	Daucosterol	C_35_H_60_O_6_	75% ethanol	Stems and leaves	[46]
**89**	Astragaloside IV	C_41_H_68_O_14_	50% (*v*/*v*) methanol–water	Aboveground parts	[16]
Alkaloids					
**90**	Indole-3-carboxaldehyde	C_9_H_7N_O	95% ethanol	Stems and branches	[45]
**91**	Indole-3-carboxylic acid	C_9_H_7N_O_2_	95% ethanol	Stems and branches	[45]
**92**	Nicotinamide	C_6_H_6N2_O	75% ethanol	Branches and leaves	[36]
**93**	4,5,4′-Trihydroxy-3,3′-iminodibenzoic acid	C_14_H_11N_O_7_	Methanol	Aboveground parts	[22]
**94**	4,5,4′,5′-Tetrahydroxy-3,3′-iminodibenzoic acid	C_14_H_11N_O_8_	Methanol	Aboveground parts	[22]
**95**	N-cinnamoylbutanediamine	C_13_H_18N2_O	Hydrochloric acid	Aboveground parts	[48]
**96**	N-cinnamidylspermidine	C_16_H_25N3_O	Hydrochloric acid	Aboveground parts	[33,48]
Other compounds					
**97**	Succinic acid	C_4_H_6_O_4_	95% ethanol	Aboveground parts	[43]
**98**	Malic acid	C_4_H_6_O_5_	Water	Branches and leaves	[33]
**99**	4-Hydroxybenzaldehyde	C_7_H_6_O_2_	95% ethanol	Stems and branches	[45]
**100**	4-Hydroxy-3,5-dimethoxybenzaldehyde	C_9_H_10_O_4_	95% ethanol	Stems and branches	[45]
**101**	Quinic acid	C_7_H_12_O_6_	Water	Branches and leaves	[33]
**102**	Phenylalanine	C_9_H_11N_O_2_	50% (*v*/*v*) methanol–water	Aboveground parts	[16]
**103**	Octanedioic acid	C_8_H_14_O_4_	95% ethanol	Stems and branches	[45]
**104**	Nonanedioic acid	C_9_H_16_O_4_	95% ethanol	Stems and branches	[45]
**105**	(E)-Dec-2-enedioic acid	C_10_H_16_O_4_	95% ethanol	Stems and branches	[45]
**106**	(E)-4-hydroxynon-2-enoic acid	C_9_H_16_O_3_	95% ethanol	Stems and branches	[45]
**107**	Palmitic acid	C_16_H_32_O_2_	95% ethanol	Aboveground parts	[43]
**108**	Pentacosanol	C_25_H_52_O	75% ethanol	Branches and leaves	[36]
**109**	Lignoceric acid	C_24_H_48_O_2_	95% ethanol	Aboveground parts	[43]
**110**	Cerotic acid	C_25_H_50_O_2_	95% ethanol	Aboveground parts	[43]
**111**	Octacosanioc acid	C_28_H_56_O_2_	95% ethanol	Aboveground parts	[43]

**Table 2:** The basic characteristics of polysaccharides in *V. coloratum*.

No	Name	Extraction Solvent	Composition	Molar Ratio	Mw (kDa)	Total Yield (%)	References
1	VCP1	Water	Glc, Gal, Ara, Rha, Man	30.6:34.3:14.9:1.7:18.5	32	15	[57]
2	VCP2	Water	Glc, Gal, Ara, GluA, GalA, Rha, Man	8.4:14.5:43.2:1.8:18.8:6.3:7.0	280	10	[57]
3	VCP3	Water	Glc, Gal, Ara, GluA, GalA, Rha, Man	5.6:10.5:33.3:1.3:31.1:13.8:4.4	21	5	[57]

Note: Man, mannose; Rha, rhamR; Rib, ribose; galA, galacturonic acid; GluA, glucuronic acid; Glc, glucose; Gal, galactose; Ara, arabinose.

**Table 3:** The basic characteristics of lectins in *V. coloratum*.

No	Name	Relative Molecular Weight of Subunits/k Da	Sugar Specificity	References
1	CM-1	27, 31	D-Galactose	[20]
2	CM-2	29, 32	D-Galactose	[20]
3	ACML-55	29, 35	D-Galactose	[59]
4	VCL	29, 35	D-Galactose	[20]
5	ML	30, 34	D-Galactose	[60]
6	CM-0	Not detected	D-Galactose	[20]

**Table 4:** Summary of the effects of *V. coloratum*.

Activity	Study design	Models	Dosages	Results	References
Anti-inflammatory effect	In vivo	Collagen-induced arthritis (CIA) mode	2 g/kg	↓ Inflammation and bone erosion, ↑ cartilage protection	[33]
	In vivo	DSS-induced colitis mode	0–200 mg/kg	↓ In vivo: DSS-induced colitis	[65]
	In vitro	Human neutrophil model	1–30 μM 1–100 μg/mL	↓ Human neutrophil proinflammatory responses	[42]
	In vivo	TNF-*α*-treated mouse model	10 mg/kg/day	↓ Atherosclerosis and inflammatory responses	[66]
	In vivo	VA-sensitized mouse model	5 mg/kg	↓ Airway inflammation and eosinophil infiltration	[68]
Anticancer effect	In vitro	HepG 2 cells	0.2, 0.4, 0.6, 0.8, 1.0 mg/mL	↑ VCP concentration, ↑ inhibition rate	[78]
	In vitro	A549 cells, NCI-H292 cells	0, 2.5, 5, 10, 20, 30, 40 μM	↑ Dose and inhibition rate	[71]
	In vitro	Twelve types of cancer cells	1~100 μmol/L	Significant therapeutic effects on lung cancer and breast cancer	[72]
	In vitro	Human osteosarcoma cells	1.25, 2.5, 5, 10, 20, 40, and 80 μg/mL	IC_50_ of *V. coloratum* >5-FU	[74]
	In vitro in vivo	Eca109 cells, MCF-7 cells, H22cells	60, 90,120 mg/kg	↑ The dose, ↑ Inhibition rate of cancer cells	[75]
Antioxidant effect	In vitro	Hydroxyl radicals, superoxide anion radicals	0.18, 0.36, 0.54, 0.72,0.90 mM. 0.06, 0.12, 0.18, 0.24, 0.30 mM.	IC_50_ values are 0.485 mM and 0.273 mM	[1]
	In vitro	DPPH and hydroxyl radical	2–10 mg/mL	↑ 2–6 mg/mL of VCP, DPPH Radical scavenging rate ↑ 2–10 mg/mL of VCP, hydroxyl radical scavenging rate	[78]
	In vitro	Hydroxyl radicals, superoxide anion radicals	100 μL	The antioxidant property of (2S)-naringenin 7-O-*β*-D-glucopyranoside is the strongest	[35]
Anti-cardiovascular disease effect	In vivo	Myocardial infarction model	15 mg/kg, 75 mg/kg	↓ Calcium influx mediated	[80]
	In vivo	Dog heart Purkinje cells, guinea pig ventricular myocytes	100 μg/mL	VCF is effective for rapid arrhythmias	[82]
	In vivo	Intracavitary mechanical injury model	100 μg/kg	↓ Proliferation of HASMCs	[83]
Other effects	In vitro	HepG2.2.15 cells	10 mg/mL	↑ VCP concentration and inhibition rate	[21]
	in vivo	Ovariectomized rat model	50 mg/kg 100 mg/kg	↓ Osteoclast activity	[15]

Note: *↑*: improved or promoted; *↓*: inhibited or reduced.

## Data Availability

No new data were created or analyzed in this study. Data sharing is not applicable to this article.

## Abbreviations

Abbreviations used, in alphabetical order:

AHR	Airway hyper-responsiveness
APA	Action potential amplitude
APD	Action potential
AQs	Aqueous extracts
BALF	Bronchoalveolar lavage fluid
CCK-8	Cell Counting Kit-8
CDDP	Cisplatin
CD	Crohn’s disease
CEEs	Crude ethanol extracts
CIA	Collagen-induced arthritis
CP	Chloroplast
EB30	1,7-Bis-(4-hydroxyphenyl)-1,4-heptadien-3-one
EtOH	Ethanol
EtOAc	Ethyl acetate
ER	Estrogen receptor
ESR	Electron spin resonance
EVC	Extract of *Viscum coloratum* (Komar.) Nakai
FMLP	Formyl-L-methionyl-L-leucyl-L-phenylalanine
HBV	Hepatitis B virus
Hedt-I	Homoeriodictyol-7-O-*β*-D-apiosiyl-(1→5)-*β*-D-apiosyl-(1→2)-*β*-D-glycoside
Hedt-II	Homoeriodictyol-7-O-*β*-D-apiose (1→2)-*β*-D-glycoside
Hedt-III	Homoeriodictyol-7-O-*β*-D-glycoside
Hedt-IV	Homoeriodictyol
Httf	5-hydroxy-3,7,3′-trimethoxyflavone-4′-O-*β*-D-glucoside
IBD	Inflammatory bowel disease
Isor	Isornetin-3-O-*β*-D-glucoside
ITRAQ	Isobaric tags for relative and absolute quantitation
MDP	Maximum diastolic potential
ML	Maximum likelihood
MMP-2	Matrix metalloprotease-2
MMP-9	Matrix metalloprotease-9
MONO	Monomer
MIX	Mixture
MI	Ischemia
MTX	Methotrexate
MTT	Methylthiazolyldiphenyl tetrazolium bromide
OVA	Ovalbumin
PAF	Fast response action potential
Pes	Petroleum ether extracts
PPE-SVC	Partially purified extract of *Viscum coloratum* (Komar.) Nakai
PKA	Protein kinase A
PTH	Parathyroid hormone
P1A	1-Phase amplitude
RA	Rheumatoid arthritis
Rham-I	Rhamnazin-3-O-*β*-D-glucoside
Rham-II	Rhamnazin-3-O-*β*-D-(6”-*β*-hydroxy-*β*-methyglutaryl)-glucoside
Rham-III	Rhamnazin-3-O-*β*-D-(6”-*β*-hydroxy-*β*-methyglutaryl)-*β*-D-glucoside-4′-O-*β*-D-glucoside
Syri	Syringin
U2OS	Human osteosarcoma cells
UC	Ulcerative colitis
VCE	Ethanol extract of *Viscum coloratum* (Komar.) Nakai
VCF	*Viscum coloratum* (Komar.) Nakai flavonoids
VCP	*Viscum coloratum* (Komar.) Nakai polysaccharides
*V. coloratum*	*Viscum coloratum* (Komar.) Nakai
*V. album* L.	*Viscum album* L.
ZO-1	Zonula occludens-1
5-FU	5-Fluorouracil
